# Treatment Adherence among HIV and TB Patients Using Single and Double Way Mobile Phone Text Messages: A Randomized Controlled Trial

**DOI:** 10.1155/2022/2980141

**Published:** 2022-08-13

**Authors:** Odette Dzemo Kibu, Vincent Verla Siysi, Same Ekobo Albert Legrand, Elvis Asangbeng Tanue, Dickson Shey Nsagha

**Affiliations:** ^1^Department of Public Health and Hygiene, University of Buea, Buea, Cameroon; ^2^Department of Medicine, University of Buea, Buea, Cameroon

## Abstract

**Background:**

Research has shown that patients fail to adhere to ART and TB treatment due to the long duration of both therapies, side effects, and forgetfulness.

**Objective:**

To assess the role of the double-way and single-way SMS on adherence to HIV and TB treatment.

**Materials and Methods:**

A randomized controlled trial among adult HIV and TB patients on treatment at the Buea Regional and Kumba District Hospitals, South West Region, Cameroon, was conducted. Participants were randomly allocated to the control, single-way, and double-way SMS intervention groups. HIV and TB participants were followed independently for a period of 6 months and 3 months, respectively. Baseline and post-intervention data were collected and analyzed using the chi-square and Student's *t*-tests with statistical significance set at *p* < 0.05.

**Results:**

A total of 210 HIV participants and 84 TB were recruited into the study with a mean age of 41.25 ± 10 years and 37.89 ± 13.27 years, respectively. Optimal adherence to ART and TB treatment at baseline was [65 (31%) CI: 0.247–0.372] and [35 (41.7%) CI: 0.311-0.522], respectively, and after the intervention, it increased to [72 (42.6%) CI: 0.347-0.495] and 41 (61.2%), respectively. There was an increase in adherence to ART among participants in the double-way SMS intervention group from 23 (32.9%) (RR: 1.04, CI: 0.8-1.31, *p*=0.716) to 29 (48.3%) (RR: 1.06, CI: 0.75-1.50, *p*=0.746). Combined single-way and double-way SMS significantly increased adherence to ART.

**Conclusions:**

The level of adherence was low among HIV and TB participants. The single-way and double-way SMS did not significantly increase adherence. However, a combination of both the double-way and single-way SMS intervention significantly improved adherence to ART.

## 1. Introduction

Adherence to antiretroviral therapy (ART) is the second strongest predictor of progression to AIDS and death, after CD4 count [1–3]. In 2017, 21.7 million people were receiving ART globally [4]. Linkage of HIV patients to care and treatment is a major challenge in achieving the 90-90-90 objective set by the United Nations Programs for AIDS (UNAIDS). As recommended by the World Health Organization (WHO), directly observed therapy (DOT) is used as the strategy for improving the treatment outcome of care for tuberculosis (TB) patients.

Suboptimal adherence to ART and anti-TB medication is a major contributing factor to treatment failure and the control and prevention of these two infectious diseases of poverty. Non-adherence to ART and anti-TB treatment might lead to an increased risk of drug resistance and prolonged infectiousness, disease relapse in the case of TB infection and even death for both [5].

The World Health Organization (WHO) defines medication adherence as the degree to which a patient's behavior corresponds with the agreed recommendations from a healthcare provider or as the extent to which the patient's history of therapeutic drug-taking coincides with the prescribed treatment [6, 7]. Studies have shown that optimal adherence to either HIV or TB is due to the prolonged duration of both therapies, side effects, and forgetfulness.

Diverse strategies or interventions have been put in place to improve adherence to ART and TB treatment one of which is the use of mobile health (mHealth) [8].There is an increased use of mobile phones in sub-Saharan Africa and the world today [9], and the administration of health care services has taken advantage of the use of phones to provide services to its patients. Mobile phones have been shown to improve healthcare delivery [10], especially in the aspect of helping patients adhere to their treatment through the use of short message service (SMS) reminders [11, 12]. The WHO strongly recommends the use of SMS for promoting adherence to treatment as part of a package of adherence interventions [13]. A lot of research has been carried out on the effectiveness of SMS to improve health care, but its effectiveness has not been tested in the South West Region of Cameroon, a conflict zone. The ongoing Anglophone crisis in the South West Region of Cameroon has brought forth a complex and challenging situation in the provision of healthcare services, one of which is the HIV and TB treatment and care programs. This crisis has inhibited access to medication and psychosocial support due to the closure of some health facilities and the supply of other hospital requirements. Due to insecurity, patients tend to boycott treatment centers. Hence, adherence to HIV and TB care and treatment becomes a challenge. Although the WHO recommends the use of the SMS intervention to improve health outcomes, the impact of SMS intervention on adherence among HIV and TB patients in the South West Region of Cameroon remains unclear. The objective of this study is to assess the role of double-way (sent message and reply received) and single-way (sent message but no reply) SMS on adherence to HIV and TB treatment.

## 2. Materials and Methods

The study protocol including the methods section has been published as a preprint in ResearchGate and as an article in the Journal of Medical Internet Research [14, 15].

### 2.1. Study Design and Setting

This study was a randomized controlled trial carried out in the South West Region of Cameroon at the Buea Regional and Kumba District Hospitals. These are state-owned hospitals accredited with HIV and TB treatment centers. These hospitals accommodate patients from other areas of the South West Region, providing health services to more than 10,000 inhabitants from this part of the country [15].

### 2.2. Participants

This study was conducted among both male and female HIV and TB participants of treatment age ≥21 years old. To qualify for inclusion in the study, participants needed a confirmed diagnosis of HIV and had to currently be on ART for at least one month, while TB participants had a confirmed diagnosis and had been on treatment for at most 3 months. The Chan method for calculating sample size for a randomized controlled trial [16] was used as shown below.

#### 2.2.1. For HIV Participants

(1) $$\begin{array}{c} n\left[\text{size per group}\right]=cX\left[\frac{p1\left(1-p1\right)+p2\left[1-p2\right]}{{\left(p1-p2\right)}^{2}}\right], \end{array}$$

where *c* = 7.9 for 80% power, and p^1^ and p^2^ are the proportion estimates (60% for the control group and 82% for the intervention group).

(2) $$\begin{array}{c} n\left[\text{size per group}\right]=7.9X\left[\frac{0.6\left(1-0.6\right)+0.82\left[1-0.82\right]}{{\left(0.6-0.82\right)}^{2}}\right],\\ n\left[\text{size per group}\right]=63.27. \end{array}$$

It was assumed that 30% of the participants would drop out of the study due to loss to follow-up and mortality. A total of 82 participants were then randomized to each group to allow for the 30% dropout (30% of 63 participants = 19). In order to make up for the 19 that were assumed to be lost to follow-up, we added the 19 participants to the calculated 63 participants, giving a total of 82 participants. Therefore, a minimum of 246 participants were recruited for the study [15].

The above sample size in each group was distributed with 164 in the intervention group (82 in the two-way SMS group and 82 in the one-way SMS group) and 82 in the control group [15].

#### 2.2.2. For TB Participants

(3) $$\begin{array}{c} n\left[\text{size per group}\right]=cX\left[\frac{p1\left(1-p1\right)+p2\left[1-p2\right]}{{\left(p1-p2\right)}^{2}}\right], \end{array}$$

where *c* = 7.9 for 80% power, and p1 and p2 are the proportion estimates (60% for the control group and 85% for the intervention group) (162).

(4) $$\begin{array}{c} n\left[\text{size per group}\right]=7.9X\left[\frac{0.6\left(1-0.6\right)+0.85\left[1-0.85\right]}{{\left(0.6-0.85\right)}^{2}}\right],\\ n\left[\text{size per group}\right]=46.45. \end{array}$$

It was assumed that 10% of the participants shall drop out of the study due to loss to follow-up and mortality. Sixty-nine participants were randomized to each arm to allow for the 10% dropout. Therefore, the required sample size shall be 153 (51 × 3 = 153) participants.

Therefore, the required sample size was 153 TB participants. The above sample size in each group was distributed as follows: 102 in the intervention group (51 used the double-way SMS approach and 51 used the single-way SMS approach) and 51 in the control group.

### 2.3. Intervention

The intervention used in this study was mobile phone text messages. Two types of SMS interventions were used; the single way (healthcare provider sends messages and expects no reply from the patient) and the double way (healthcare provider sends messages and expects a reply from the patient). This intervention was based on the principle of the health belief model. HIV and TB participants were randomly assigned to the intervention groups and control groups. The control group received only the treatment and the standard care provided at the treatment centers [15].

### 2.4. Intervention among HIV Participants

#### 2.4.1. Single-Way SMS

The single-way SMS intervention was to remind the participants to take his/her prescribed medication. Participants who replied to the SMS sent by the researcher were excluded from the study. The text messages were sent for a period of 6 months. Air time was purchased from a telephone network company. Messages were sent thrice a week (Mondays, Wednesdays, and Fridays) [15].

#### 2.4.2. Double-Way SMS

The double-way SMS was also used as an intervention to remind the participants to take their prescribed medication. Participants in this intervention arm were obliged to reply to the SMS sent by the researcher. The text messages were sent for a period of 6 months. Air time was purchased from a telephone. Messages were sent thrice a week (Mondays, Wednesdays, and Fridays) [15].

### 2.5. Intervention among TB Participants

The two interventions used among the TB participants were the same single- and double-way SMS for a period of 3 months.

### 2.6. Development of the Intervention

The development phase of the application involved continuous consultations with HIV and TB patients and healthcare providers within the specific context and literature reviews. These processes involved key informant interviews and focus group discussions to inform the intervention and refine the features, as well as to ensure project buy-in and identify relevant contextual barriers and conditions to facilitate the intervention.

### 2.7. Enrollment and Randomization

**Figure 1:**
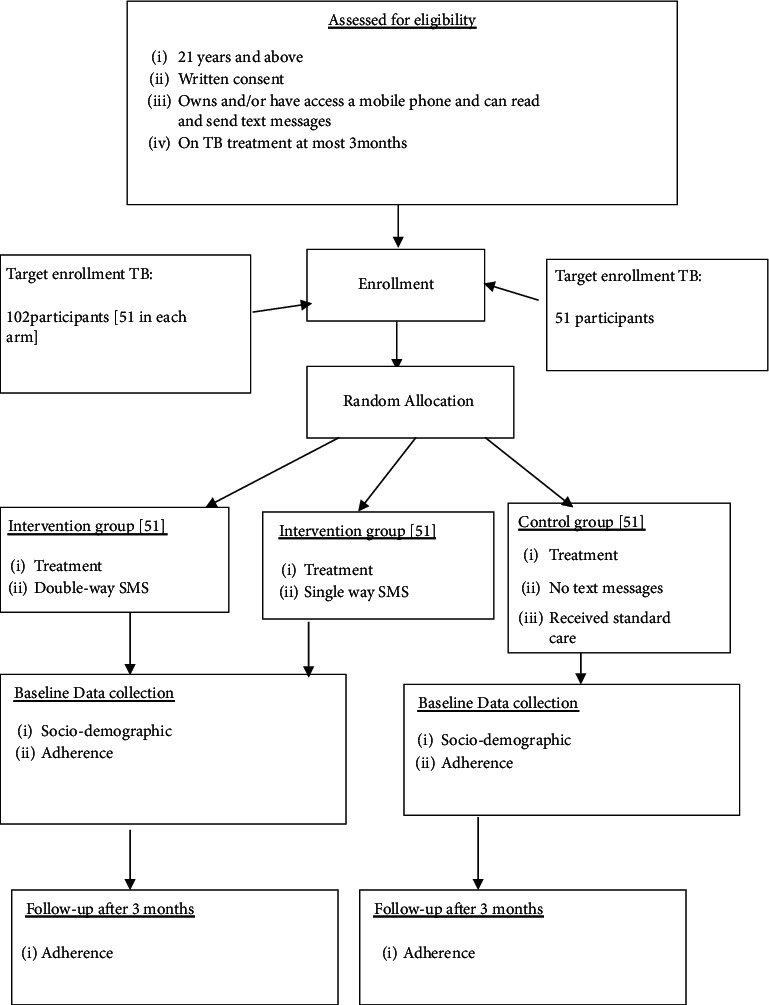
Flow chart showing recruitment and random allocation of the TB participants in the intervention and control groups.

**Figure 2:**
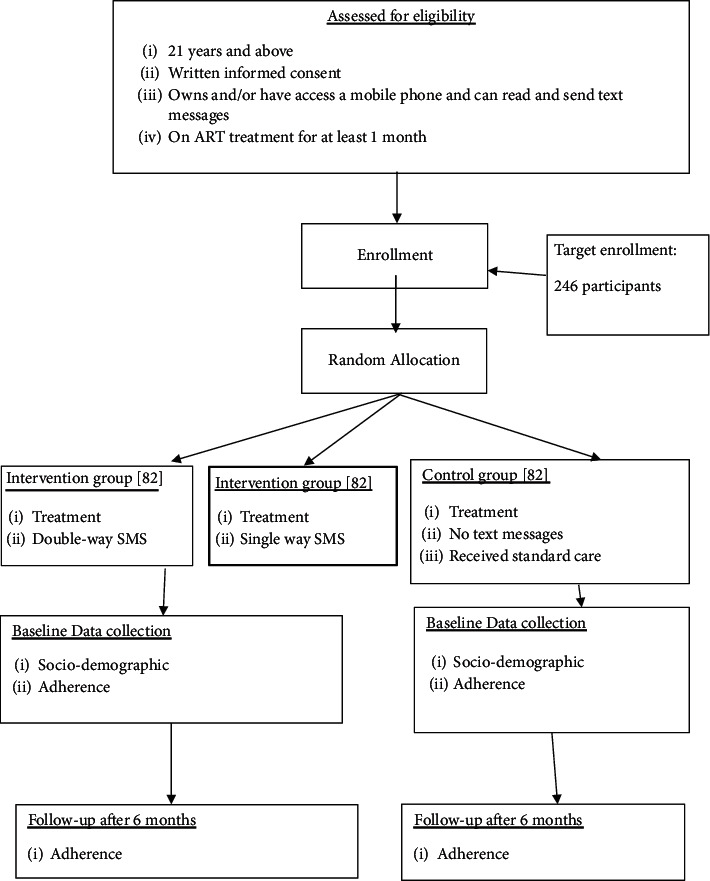
Flow chart showing recruitment and random allocation of the HIV participants in the intervention and control groups [15].

tVoluntary informed consent was collected from each study participant after screening for eligibility and randomization of the intervention and control areas. Participants were randomly allocated in a 1-to-1-to-1 ratio into the double-way and single-way SMS intervention and control groups. The randomization blocks were generated and used following the allocation concealment procedure where the participants, data collectors, and statisticians were not aware of the allocation sequences prior to randomization. Figures 1 and 2 show the random allocation of HIV and TB participants in the intervention groups and the control group.

### 2.8. Evaluation of Intervention

A logbook was used to register all SMS text messages sent and replies received on the designated days of the week. A delivery report function was used to ensure that the messages were delivered to the participants. The sent text and responses received were evaluated by calculating the proportion of SMS text messages sent and responses received [15].

Examples of the text messages that were sent.

“Hello. Do remember to take your medication as prescribed. Your health is your wealth.”

“You are kindly reminded to take your medication. Your health is our priority.”

“Don't forget to take your medications.”

Examples of the text messages received.

Okay Thank you. I will.

### 2.9. Ethical Consideration

Ethical approval was obtained from the Faculty of Health Sciences Institutional Review Board (reference number: 2018/764-03). Participants signed a consent form before their enrollment into the study. Participation in the study was voluntary.

### 2.10. Data Collection

The overall adherence of each participant was measured using 2 scales of adherence measurement, namely, the visual analogue scale (VAS) and the Center for Adherence Support Evaluation (CASE) adherence index. A composite score for adherence was derived from these 2 scales of adherence [15].

### 2.11. Data Management and Analysis

Information from the questionnaire was checked each time it was brought from the field for unfilled and unanswered questions and edited for the use of correct codes by the principal investigator. A code was given to identify each participant. The data were analyzed following the guidelines for Consolidated Standards of Reporting Trials (CONSORT). Baseline characteristics were presented separately for each randomized group. Descriptive statistics were used to summarize participants' demographics and to assess the baseline clinical characteristics of all study participants. The *T*-test was used to compare groups on continuous outcomes, and the chi-squared test for binary outcomes. All statistical tests were performed using two-sided tests at the 0.05 level of significance.

## 3. Results

### 3.1. Distribution of HIV and TB Participants on Treatment before and after of Single-Way and Double-Way SMS Intervention

The study was able to recruit a total of 210 and 84 HIV and TB participants, respectively. Out of the 210 HIV participants on antiretroviral therapy (ART), 70 (33.3%) participants per group were randomly distributed into the control, single-way SMS, and double-way SMS groups. Likewise, out of the 84 TB participants on TB treatment (TBT) recruited into the study, 28 (33.3%) participants were randomly distributed into the control, single-way SMS, and double-way SMS groups. However, after 6 months of follow-up of HIV participants and 3 months for TB participants, 18.6% (39 out of 210) of HIV participants and 20.2% (17 out of 84) of TB participants dropped out of the study.

### 3.2. Demographic Characteristics of HIV and TB Participants on Treatment

The ages of the HIV participants ranged from 21 years and above with a mean age of 41.25 ± 10.00 years. The majority of these participants [78 (37.1%) (CI 95%: 0.306-0.437)] were between the ages of 31-40 years. Out of 210 HIV participants, the majority were made up of females [137 (65%) (CI 95%: 0.588-0.717)] as shown in Table 1. The TB participants had an equal distribution of males and females with a mean age of 37.89 ± 13.27 years. Most of the TB participants [31 (36.9%) (CI 95%: 0.266-0.472)) were between 21-30 years as shown in Table 1.

### 3.3. Clinical Characteristics of HIV and TB Participants on ART and TB Treatment

Table 2 shows the clinical characteristics of HIV and TB participants on treatment. Among the different HAART regimens administered to the participants, the tenofovir lamivudine and efvavirenz combination recorded the highest frequency regimen that was administered to the participants. The majority of the participants (109 (51.9%) (CI 95%: 0.451-0.587)) had been on treatment for between 1-4 months. None of the participants were co-infected with TB; however, 9 (4.29%) (CI 95%: 0.015–0.070) of the participants were infected with other diseases as shown in Table 2.

Among the TB participants, some were (46 (54.8%) (CI 95%: 0.441-0.654)) were co-infected with HIV, and (28 (33.3%) (CI 95%: 0.333–0.434) were infected with other diseases. The majority of the TB participants (63 (75.0%) (CI 95%: 0.657-0.843)) were within the first 3 months of treatment as shown in Table 2.

### 3.4. Adherence to Treatment and Demographic Characteristics among the HIV and TB Participants on Treatment from Buea and Kumba Hospitals

Table 3 shows that there was no association between the demographic characteristics and adherence to ART. Compared to males, the majority of the females (91 (66.4%)) had poor adherence. However, there was no statistically significant difference between poor and good adherence with respect to gender. HIV participants who were between 31-40 years had the highest optimal adherence 20 (25.6%) compared to the participants in the other age groups.

Generally, there was no association between demographic characteristics and adherence to TB treatment as shown in Table 4. However, females had a higher percentage (26 (61.9%)) of poor adherence compared to their male counterparts 23 (54.8%). TB participants who were males, aged between 21-30 years, had a secondary level of education or above and singled had better optimal adherence to treatment.

### 3.5. Clinical Characteristics and Adherence among HIV and TB Participants on Treatment from the Buea and Kumba Hospitals

Most of the participants on ART were on the first line of treatment, and they had a higher proportion of poor adherence to ART 136 (69.4%) than those who were on the second line of treatment. However, there was no statistically significant difference between those who were on the first-line compared to those who were on the second-line with respect to their adherence to ART. HIV participants who were not infected with TB had a statistically significant higher proportion of non-adherence 118 (69.4) than those who were infected with TB (Table 5).

Among the TB participants, those who were co-infected with HIV, co-infected with other diseases, and the duration of TB treatment had no statistical association with adherence as shown in Table 6.

### 3.6. Overall Adherence to ART at Baseline and after the Single-Way and Double-Way SMS Intervention Using the Different Adherence Measurement Scales

**Figure 3:**
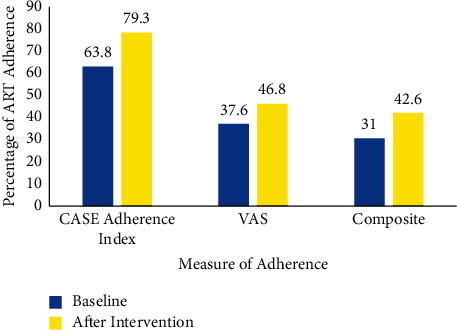
Level of adherence to ART among PLWHA as measured by VAS and CASE adherence index at baseline and after the Single way and Double way SMS Intervention. Key: VAS; Visual Analogue Scale, CASE: Center for Adherence Support Evaluation.

tAdherence to ART was measured using 2 scales of measurement, namely, the Visual Analogue Scale (VAS) and the Center for Adherence Support Evaluation (CASE) adherence index. Using the CASE Adherence index, HIV participants reported a higher percentage of 134 (63.8%) of optimal adherence than VAS 79 (37.6%) at baseline (Figure 3). The composite score for adherence was 65 (31%).

After 6 months of intervention, the CASE Adherence index still reported a higher percentage of 134 (79.3%) of adherence than VAS 80 (46.8%) (Figure 3). The composite score for adherence was higher than 72 (42.6%) after the intervention.

### 3.7. Overall Adherence to TB Treatment at Baseline and after the Single-Way and Double-Way SMS Intervention Measured by Different Adherence Scales

**Figure 4:**
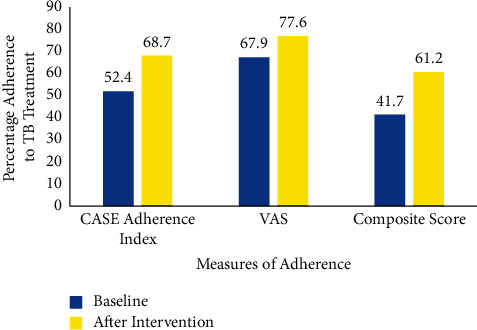
Level of adherence to DOTS TB participants as measured by VAS and CASE adherence index at Baseline and after the single-way and double-way SMS intervention. Key: VAS; Visual Analogue Scale, CASE: Center for Adherence Support Evaluation.

In Figure 4, the optimal adherence for TB patients increased from 35 (41.7%) to 41 (61.2%). The visual analog scale recorded a higher proportion of participants with optimal adherence at baseline and also after the intervention.

### 3.8. Adherence to ART among the Participants in the Single-Way and Double-Way SMS and the Control Groups

At baseline, participants in the control group and the single way SMS (SWS) group had an equal number of participants with poor adherence 49 (70%) and good adherence 21 (30%). There was no statistically significant difference between the groups (*P*=1.000). After the intervention, participants in the SWS group showed an increase in the level of good adherence 26 (44.8%) (Table 7).

In the double-way SMS (DWS) group at baseline, 47 (67.1%) participants had poor adherence compared to the control group 49 (70.0%). At baseline, participants in the control group had a slightly higher risk (1.04) of having poor adherence than participants in the DWS group. However, there was no statistically significant difference (*P*=0.716). After the intervention, the participants in the DWS had a higher level of adherence to ART compared to the control group. A relative risk of 1.06 was recorded between the control and DWS groups which was not statistically significant (*P*=0.746) (Table 7).

### 3.9. Adherence to TB Treatment among the Participants in the Single-Way and Double-Way SMS and the Control Groups

At baseline, participants in the control group had a higher number of participants with poor adherence [20 (71.4%)] than to participants in the SWS and also to participants in the DWS group. The relative risk of 1.43 and 1.33, respectively, showed no statistically significant difference between the two groups (*p*=0.101 and 0.168, respectively) (Table 8).

After the intervention, participants in the control group showed an increase in the level of good adherence 16 (80%) when compared to participants on DWS with a relative risk of slightly lower than 1 which was statistically significantly different (*P*=0.039) (Table 8).

### 3.10. Combined Single-Way and Double-Way Adherence to ART

Participants on ART in the intervention groups (SWS and DWS) were pooled into a single group, and the pooled adherence was assessed at baseline and after the intervention. At baseline, 96 (68.6%) participants had a poor level of adherence compared to 44 (31.4%) participants with good adherence. After the intervention, the number of participants with a good adherence level increased to 55 (46.6%) with a statistically significant difference of 0.012 (Table 9).

## 4. Discussion

### 4.1. Importance of the Study to Healthcare Delivery

The use of mobile phones as a tool to measure adherence is becoming universal. The introduction and use of mobile health (mHealth) initiatives are variable and depend on the settings [17–19]. This study was carried out to assess the role of a single-way and double-way SMS in improving adherence among HIV and TB patients to treatment. This study is of great public health importance because it comes at a time when the WHO is promoting the use of mHealth to improve healthcare delivery. The findings from this research are important to help guide healthcare providers and policymakers on how and when to use SMS to improve adherence.

### 4.2. Characteristics of the HIV and TB Study Population

It was observed from the study that the majority of the HIV and TB participants were between 31-40 years and 21-30 years, respectively, which represents the working force of an economy and thus, posing a threat to the growth and future of the economy of Cameroon. A study in Nigeria reported the mean age of patients as 35.6 years infected with HIV, and 75% of the patients were in the age bracket of 20-49 years.

Women demonstrated good adherence to ART meanwhile the men showed good adherence to TB treatment (TBT). This may be due to the fact that women are always involved in psychosocial support groups where they find it easy to adhere to treatment. On the other hand, the good adherence observed among males may be attributed to the fact that women are more concerned about the stigma related to TB and may boycott coming to the hospital for routine treatment. No significant difference compared to the opposite sex in each group was recorded. Data from the study carried out by Fonsah and colleagues in 2017 in Yaoundé, Cameroon [20], showed that males had a lower proportion of good adherence than females (76.67% vs. 90.84%) due to the side effects of the drug or having missed their doses. However, other studies have shown that females are less likely to adhere to treatment such as in a cohort study carried out in 2013 in Brazil [21] by Palmira and colleagues established from their findings that the incidence of non-adherence to ART was 1.5 times greater among women than men. A similar study carried out by Tapp and colleagues in 2011 showed that female injection drug users were less likely to access and adhere to antiretroviral therapy [22].

We found out from the study that HIV participants who had been infected with TB had a significant decrease in adherence compared to those who had not been infected with TB. This can be explained by the fact that poor adherence to ART exposes patients to opportunistic infection and the commonest being TB. A study carried out by Fonsah and colleagues in Cameroon showed that HIV patients with opportunistic infections had 3.1-times higher odds of having been non-adherent (*p* < 0.0003), with significantly longer periods of non-adherence, compared to subjects without opportunistic infections (*p*=0.02) [20]. On the other hand, it was observed that 38 (45.2%) of the TB participants were infected with HIV and showed a non-significant poor adherence to TB treatment compared to TB participants who were not infected with HIV.

### 4.3. Impact of Single-Way SMS on Adherence to HIV and TB Treatment from the Intervention Study

The study found out that at baseline, participants in the control group and single-way SMS group have the same percentage of good adherence of 21 (30.0%). After the intervention (single-way SMS), the HIV participants had a higher percentage of good adherence than the control. However, this difference was not significant. Studies have revealed that the impact of SMS programs on adherence has been studied in multiple randomized controlled trials (RCTs), and SMS intervention has generally demonstrated positive results. Two RCTs of weekly SMS programs in Kenya, using local languages for newly initiating ART patients, demonstrated the effectiveness of the SMS programs [14, 23]. This intervention proved effective in Kenya. The difference in the findings from our study might be due to the fact that the researchers from Kenya used the local language in the content of the SMS. A similar finding to the study carried out in Kenya was established in Nigeria [24] through an RCT involving adherence counseling plus twice-weekly SMS among initially non-adherent subjects. The result of the study showed a significant improvement in self-reported adherence. In addition, an RCT carried out in Cameroon by Bigna and colleagues in 2014 examined SMS appointment reminders for caregivers of children living with HIV found that the SMS significantly improved appointment attendance compared to the standard of care [25]. This study carried out in Cameroon only helped the caregivers to respect appointment dates, and it is not possible to conclude that SMS helped the children adhere to treatment after the caregivers have respected appointment dates.

At HIV treatment centers calls and SMS are used by healthcare providers to remind patients to come for drug refills but this method does not suffice for us to conclude that SMS helps patients to adhere to treatment. The single-way SMS did not show a significant effect on improving adherence. This can be due to the current socio-political in the North West and South West Regions of the country. Although existing literature has shown that SMS significantly improves adherence, our study shows that SMS intervention will not be effective if the setting where the SMS is to be implemented is not tested. A study carried out by Nsagha and colleagues established that HIV/TB co-infected patients who were on standard care and received SMSs adhered to their ART [26]. However, SMS was found to have no effect on adherence. Another RCT in carried out by Mbuagbaw and colleagues in 2012 in Cameroon reported that using weekly SMS for patients on ART found no significant benefit on multiple adherence metrics [27].

### 4.4. Impact of Double-Way SMS on Adherence to HIV and TB Treatment from the Intervention Study

This approach of SMS was used to test its effectiveness in improving adherence to the traditional single-way SMS. This study showed that the double-way SMS increased the adherence of HIV patients compared to the control. However, this increase was not significant. The non-significance of the results might be due to the fact that some of the participants did not reply to the text messages on the same day they received the text or because the routine activity of replying to text messages overburdened them. A systematic study carried out by Velthoven [28] and colleagues were carried out on mobile phone messaging for HIV prevention, appointment reminders, HIV testing reminders, medication adherence, and communication between healthcare workers. Of the three randomized controlled trials assessing the use of short message service (SMS) to improve medication adherence, two showed positive results. Other interventional studies did not provide significant results. They concluded that despite an extensive search, limited evidence was found on the effectiveness of mobile phone messaging for HIV care.

Both the single-way and double-way SMS did not show significant improvement in adherence among the participants on TB treatment. This might be explained by the fact that adherence measures were done after three months of treatment and the measure was done once. This is in line with a study carried out in Pakistan by Mohamed and colleagues in 2016 [29] who demonstrated the fact that there was no significant program effect on self-reported medication adherence reported among the TB participants on treatment after they had responded to SMS sent. The finding from our study was also similar to the findings of Liu and colleagues in 2015 in China [30] who showed that two-way SMS reminders had no impact on medication adherence for tuberculosis patients.

In addition, a study carried out by Bediang and colleagues in 2014 in the Center Region of Cameroon also showed that treatment adherence did not improve after patients received reminders SMS for a period of 6 months, and a lower treatment rate for TB was observed [17]. Their study corroborated the results of a study exploring the acceptance and perception of SMS use to improve TB treatment compliance [31]. However, sending only SMS denotes a “one-way” communication which is contradictory to effective patient education. They suggested an interactive mhealth combining SMS reminders, sent to patients by the healthcare staff, with the possibility given to patients to submit their own questions and concerns back to the healthcare professionals.

These findings from our study contradict findings from existing literature on the effectiveness of SMS on adherence, which has led the WHO to recommend the use of SMS in healthcare settings. However, our contrary results might be due to the fact that they might be a trend of greater effect with text messages that are personalized, and the frequent medication reminders result in user fatigue. Whiles some experts suggest that the most important factor is whether patients feel cared for, not the length or frequency of the text messages. Perhaps the lack of more personalized engagement, the didactic nature of our messages, and the SMS message being received when the patient was not in close proximity to his/her medication all contributed to the failure to reduce poor adherence [30].

Nicole and colleagues in an article mentioned that medication adherence is often subject to multilevel influences beyond the individual and investigating mechanisms of action through which interventions may promote this behavior facilitates the delivery of systematically created and targeted approaches [32]. Specifying linkages between intervention functions and influences on the target behavior reveals appropriate strategies for potentially bringing about change [33]. A complementary behavior change taxonomy further elaborates upon these intervention functions in terms of many specific behavior change techniques (BCTs) [34]. Moreover, a Behavior Change Wheel (BCW) highlights the importance of taking into account multilevel influences on behavior through the incorporation of the Capability Opportunity Motivation-Behavior (COM-B) model of behavior. The COM-B model states that capability is related to one having the psychological (required knowledge and skills) and physical capacity to engage in the behavior. Opportunity is divided into physical (associated with the external environment) and social (i.e., cultural norms and interpersonal influences) factors that promote or impede behavior within the individual [33]. Motivation encompasses all brain activity that provokes behavior, including automatic (i.e., emotions, wants and needs, and habits) and reflective (i.e., belief systems, plans, and evaluations) processes [33]. It is important to elaborate on the BCTs of an intervention that can bring about a change in behavior. There are 8 BCTs that have been identified in the 2-way communication intervention. However, our study identified 2 BCTs which were the prompt cue to action. This technique affects only the physical aspect of the opportunity and the automatic aspect of the motivation belonging to the COM-B model. In addition, our study did not implement interactive double-way communication. These are some of the reasons why the double-way SMS intervention alone did not change the behavior of the HIV participants in helping them adhere to their treatment.

## 5. Conclusion

Although, the single-way SMS and the double-way SMS intervention did not significantly improve adherence among the HIV and TB participants on treatment, a combination of both the double- and single-way SMS improved adherence to ART. It is important to assess the setting before using the SMS intervention as a strategy to improve adherence.

## Figures and Tables

**Table 1:** Demographic characteristics of TB and HIV participants in Buea and Kumba hospitals.

Demographic characteristics		HIV	TB
		No (%)	No (%)
Gender	Male	73 (34.8)	42 (50.0)
	Female	137 (65.2)	42 (50.0)
	Total	**210 (100)**	**84 (100)**

Age group (Years)	21-30 years	34 (16.2)	31 (36.9)
	31-40 years	78 (37.1)	27 (32.1)
	41-50 years	58 (27.6)	13 (15.5)
	>50 years	40 (19.0)	13 (15.5)
	Total	**210 (100)**	**84 (100)**

Educational level	No education	13 (6.2)	1 (1.2)
	Primary	90 (42.9)	37 (44.0)
	Secondary	90 (42.9)	37 (44.0)
	Tertiary	17 (8.1)	9 (10.7)
	Total	**210 (100)**	**84 (100)**

Marital status	Single	77 (36.7)	41 (48.8)
	Married	83 (39.5)	35 (41.7)
	Divorced	16 (7.6)	2 (2.4)
	Widowed	34 (16.2)	6 (7.1)
	Total	**210 (100)**	**84 (100)**

**Table 2:** Clinical characteristics of HIV and TB participants on treatment in the Buea and Kumba Hospitals.

Clinical characteristics		HIV	TB
		No (%)	No (%)
Treatment regimen	TeLe	185 (88.10)	—
	TeLeNev	3 (1.43)	—
	ZiLNev	8 (3.81)	
	TeL + PI	11 (5.24)	—
	Dou + EFV	3 (1.43)	—
	Total	**210 (100)**	

Duration on HAART (Months)	1–7	159 (75.7)	—
	>7	51 (24.29)	—
	Total	**210 (100)**	

Duration on TB treatment (Months)	1-3	—	63 (75.0)
	4	—	21 (25.0)
	Total		**84 (100)**

Co-infection with TB	No	210 (100)	—
	Yes	0 (0)	—
	Total	**210 (100)**	

Co-infection with HIV	No	—	46 (54.8)
	Yes	—	38 (45.2)
	Total		**84 (100)**

Other diseases (malaria, intestinal parasitic infections	No	201 (95.71)	56 (66.7)
	Yes	9 (4.29)	28 (33.3)
	Total	**210 (100)**	**84 (100)**

Key: Tele: tenofovir, lamivudine, efavirenz; TelNev: tenofovir, lamivudine, nevirapine; ZiLNev: zidovudine, lamivudine, nevirapine; TeL + PI: tenofovir, lamivudine + protein inhibitor; Dou + EFV; NCDs:non-communicable diseases.

**Table 3:** Adherence to ART and demographic characteristics among the participants from Buea and Kumba Hospitals.

Demographics characteristics		Adherence status			
		Poor	Good	*χ* ^2^	*P*-value
		No (%)	No (%)		
Gender	Male	54 (74.0)	19 (26.0)	1.427	0.260
	Female	91 (66.4)	46 (33.6)		
	Total	145 (69.0)	65 (31.0)		

Age group (Years)	21-30 years	23 (67.6)	11 (32.4)	1.952	0.582
	31-40 years	58 (74.4)	20 (25.6)		
	41-50 years	39 (67.2)	19 (32.8)		
	>50 years	25 (62.5)	15 (37.5)		
	Total	145 (69.0)	65 (31.0)		

Educational level	Primary and below	87 (72.5)	33 (27.5)	1.562	0.211
	Secondary and above	58 (64.4)	32 (35.6)		
	Total	145 (69.0)	65 (31.0)		

Marital status	Singled	88 (69.3)	39 (30.7)	0.009	0.925
	Married	57 (68.7)	26 (31.3)		
	Total	145 (69.0)	65 (31.0)		

**Table 4:** Adherence to TB treatment and demographic characteristics among the participants from Buea and Kumba Hospitals.

Demographic characteristics		Adherence status			
		Poor	Good	*χ* ^2^	*P*-value
Gender	Male	23 (54.8)	19 (45.2)	0.441	0.507
	Female	26 (61.9)	16 (38.1)		
	Total	**49 (58.3)**	**35 (41.7)**		

Age group (Years)	21-30 years	14 (45.2)	17 (54.8)	3.674	0.299
	31-40 years	18 (66.7)	9 (33.3)		
	41-50 years	9 (69.2)	4 (30.8)		
	>50 years	8 (61.5)	5 (38.5)		
	Total	**49 (58.3)**	**35 (41.7)**		

Education	Primary and below	25 (65.8)	13 (34.2)	1.587	0.208
	Secondary and above	24 (52.2)	22 (47.8)		
	Total	**49 (58.3)**	**35 (41.7)**		

Marital status	Singled	27 (55.1)	22 (44.9)	0.505	0.477
	Married	22 (62.9)	13 (37.1)		
	Total	**49 (58.3)**	**35 (41.7)**		

*χ* ^2^: Chi square.

**Table 5:** Adherence to ART treatment and clinical characteristics among participants from the Buea and Kumba Hospitals.

Clinical characteristics		Adherence status			
		Poor	Good	*χ* ^2^	*P*-value
		No (%)	No (%)		
HAART regimen	First line	136 (69.4)	60 (30.6)	0.159	0.690
	Second line	9 (64.3)	5 (35.7)		
	Total	**145 (69.0)**	**65 (31.0)**		

Duration on HAART (months)	1-4	81 (74.3)	28 (25.7)	10.334	0.006
	5-7	38 (76.0)	12 (24.0)		
	>7	26 (51.0)	25 (49.0)		
	Total	**145 (69.0)**	**65 (31.0)**		

Past history TB Infection	No	118 (69.4)	52 (30.6)	0.055	0.814
	Yes	27 (67.5)	13 (32.5)		
	Total	**145 (69.0)**	**65 (31.0)**		

Presence of Other disease (malaria, Cryptosporidium parvum spp)	No	140 (69.7)	61 (30.3)	0.801	0.371
	Yes	5 (55.6)	4 (44.4)		
	Total	**145 (69.0)**	**65 (31.0)**		

*χ* ^2^: Chi square.

**Table 6:** Adherence to TB treatment and clinical characteristics among participants from the Buea and Kumba Hospitals.

Clinical characteristics		Adherence status			
		Poor No (%)	Good No (%)	*P*-value	
HIV co-infection	No	26 (56.5)	20 (43.5)	0.137	0.711
	Yes	23 (60.5)	15 (39.5)		
	Total	49 (58.3)	35 (41.7)		

Duration on TB treatment (Months)	**1-3**	35 (55.6)	28 (44.4)	0.800	0.371
	**4**	14 (66.7)	7 (33.3)		
	Total	49 (58.3)	35 (41.7)		

Presence of other disease (malaria, *Cryptosporidium parvum* spp)	No	31 (55.4)	25 (44.6)	0.612	0.434
	Yes	18 (64.3)	10 (35.7)		
	Total	49 (58.3)	35 (41.7)		

*χ* ^2^: Chi square.

**Table 7:** Adherence to ART between the single-way and double-way SMS and control of the groups before and after 6 months of intervention.

Assessment	Group	Level of adherence		Effect estimate	
		Poor No (%)	Good No (%)	RR (95% CI)	*p*-value
Baseline	Control	49 (70.0)	21 (30.0)	1.00 (0.81-1.24)	1.000
	Single-way SMS	49 (70.0)	21 (30.0)		
	Control	49 (70.0)	21 (30.0)	1.04 (0.83-1.31)	0.716
	Double-way SMS	47 (67.1)	23 (32.9)		

After 6 Months intervention	Control	29 (54.7)	24 (45.3)	0.99 (0.71–1.39)	0.962
	Single-way SMS	32 (55.2)	26 (44.8)		
	Control	29 (54.7)	24 (45.3)	1.06 (0.75–1.50)	0.746
	Double-way SMS	31 (51.7)	29 (48.3)		

RR = risk ratio; CI = confidence interval; SMS: short message service.

**Table 8:** Adherence to TB treatment between the single-way and double-way SMS and control of the groups before and after 3 months of intervention.

Assessment	Group	Level of adherence		Effect estimate	
		Poor No (%)	Good No (%)	RR (95% CI)	*p*-value
Baseline	Control	20 (71.4)	8 (28.6)	1.43 (0.92–2.21)	0.101
	Single-way SMS	14 (50.0)	14 (50.0)		
	Control	20 (71.4)	8 (28.6)	1.33 (0.88–2.02)	0.168
	Double-way SMS	15 (53.6)	13 (46.4)		

After 3 months intervention	Control	4 (20.0)	16 (80.0)	0.46 (0.17–1.24)	0.101
	Single-way SMS	10 (43.5)	13 (56.3)		
	Control	4 (20.0)	16 (80.0)	0.40 (0.15–1.05)	0.039
	Double-way SMS	12 (50.0)	12 (50.0)		

RR = risk ratio; CI = Confidence Interval; SMS: short message service.

**Table 9:** Combined single-way and double-way adherence to ART before and after 6 months of intervention.

		Adherence		Effect estimate	
		Poor	Good	RR (95% CI)	*p*-value
		No (%)	No (%)		
Combined groups (SWS and DWS)	Baseline	96 (68.6)	44 (31.4)	1.28 (1.05-1.57)	**0.012**
	6 months intervention	63 (53.4)	55 (46.6)		
	Total	**159 (61.6)**	**99 (38.4)**		

Control group	Baseline	49 (70.0)	21 (50.0)	1.28 (0.96-1.71)	0.081
	6 months intervention	29 (54.7)	24 (45.3)		
	Total	78 (63.4)	45 (36.6)		

SWS: single-way SMS; DWS: double-way SMS; CI: confidence interval; RR: risk ratio.

## Data Availability

All data generated and analyzed during this study are included in this manuscript. Data will be provided upon request from the corresponding author.

## Abbreviations

ATT: Anti-tuberculosis therapy
BRH: Buea regional hospital
CASE: Center for adherence support and evaluation
DWS: Double way SMS
KDH: Kumba district hospital
DOTS: Directly observed therapy, short-course
HAART: Highly active antiretroviral therapy
RCT: Randomized controlled trial
SWS: Single-way SMS
SMS: Short message service
ART: Antiretroviral therapy
TB: Tuberculosis
TBT: Tuberculosis treatment
PLWHA: People living with HIV/AIDS
VAS: Visual analogue scale
WHO: World Health Organisation.
